# Sex Is a Determinant for Deoxynivalenol Metabolism and Elimination in the Mouse

**DOI:** 10.3390/toxins9080240

**Published:** 2017-08-04

**Authors:** James J. Pestka, Erica S. Clark, Heidi E. Schwartz-Zimmermann, Franz Berthiller

**Affiliations:** 1 Department of Food Science and Human Nutrition, Michigan State University, East Lansing, MI 48824, USA; clarkerica7@gmail.com; 2 Center for Integrative Toxicology, Michigan State University, East Lansing, MI 48824, USA; 3 Department of Microbiology and Molecular Genetics, Michigan State University, East Lansing, MI 48824, USA; 4 Christian Doppler Laboratory for Mycotoxin Metabolism, Center for Analytical Chemistry, Department of Agrobiotechnology (IFA-Tulln), University of Natural Resources and Life Sciences, Vienna, 3430 Tulln, Austria; heidi.schwartz@boku.ac.at (H.E.S.-Z.); franz.berthiller@boku.ac.at (F.B.)

**Keywords:** trichothecene, mycotoxin, metabolism, sex dependence

## Abstract

Based on prior observations that deoxynivalenol (DON) toxicity is sex-dependent, we compared metabolism and clearance of this toxin in male and female mice. Following intraperitoneal challenge with 1 mg/kg bw DON, the dose used in the aforementioned toxicity study, ELISA and LC–MS/MS analyses revealed that by 24 h, most DON and DON metabolites were excreted via urine (49–86%) as compared to feces (1.2–8.3%). Females excreted DON and its principal metabolites (DON-3-, DON-8,15 hemiketal-8-, and iso-DON-8-glucuronides) in urine more rapidly than males. Metabolite concentrations were typically 2 to 4 times higher in the livers and kidneys of males than females from 1 to 4 h after dosing. Trace levels of DON-3-sulfate and DON-15-sulfate were found in urine, liver and kidneys from females but not males. Fecal excretion of DON and DON sulfonates was approximately 2-fold greater in males than females. Finally, decreased DON clearance rates in males could not be explained by glucuronidation activities in liver and kidney microsomes. To summarize, increased sensitivity of male mice to DON’s toxic effects as compared to females corresponds to decreased ability to clear the toxin via urine but did not appear to result from differences in toxin metabolism.

## 1. Introduction

**Figure 1:**
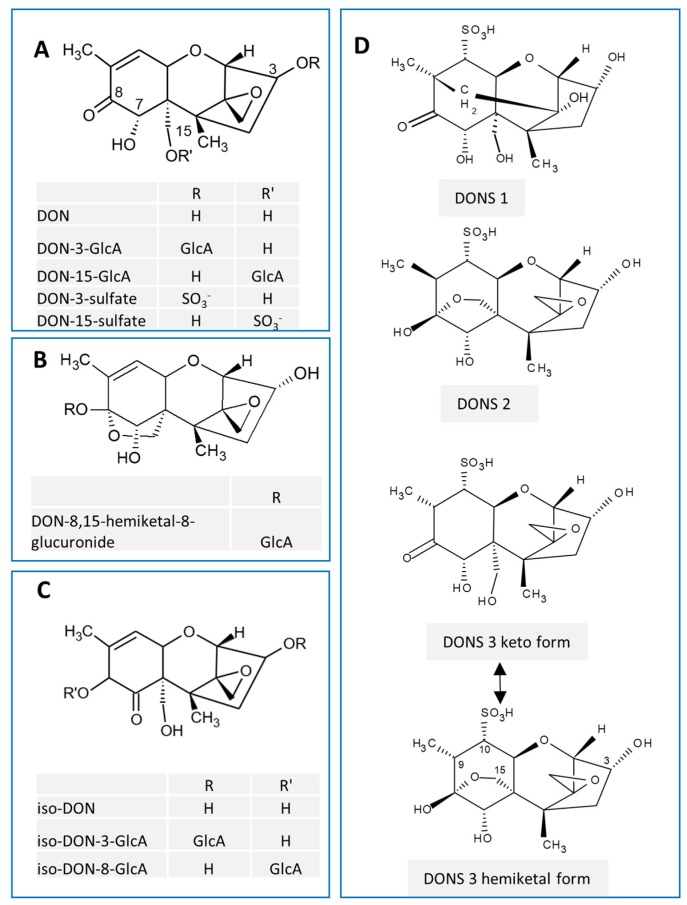
Chemical structures of major deoxynivalenol (DON) metabolites. (**A**) DON-glucuronides (DON-GlcAs) and DON-sulfates, (**B**) DON-8,15 hemiketal-8-glucuronide, (**C**) iso-DON and its glucuronides, (**D**) DON sulfonates (DONS).

Deoxynivalenol (DON, vomitoxin), a trichothecene mycotoxin mainly produced by *Fusarium graminearum*, frequently contaminates cereal crops. DON adversely impacts human and animal health by targeting the gastrointestinal tract and immune systems [1]. Metabolism of this toxin is highly-species dependent [2,3]. Regioselective glucuronidation is the major phase II pathway by which DON is metabolized. DON-15-glucuronic acid (DON-15-GlcA) and DON-3-glucuronic acid (DON-3-GlcA) are major and minor metabolites (Figure 1A), respectively, of this mycotoxin in humans [4,5]. Non-human animals exhibit DON glucuronide patterns [6] that can include DON-8,15 hemiketal-8-glucuronic acid (DON-8,15 hk-8-GlcA; Figure 1B) [7,8], iso-DON-3-glucuronic acid (iso-DON-3-GlcA, most likely previously identified as DON-7-GlcA) and iso-DON-8-GlcA (Figure 1C) [9,10]. Depending on the animal species, DON is also metabolized by sulfation and/or sulfonation. DON sulfates (Figure 1A) are DON metabolites in poultry [11,12] and have more recently been determined in human urine [13]. DON sulfonates (DONS 1, 2 and 3; Figure 1D) are major DON metabolites in rats. Schwartz-Zimmerman and coworkers [14] observed that rats orally exposed to DON excreted 48% of the total DON dose as DON- and deepoxy-DON sulfonates in feces [11].

Deepoxy-DON (9,12-diene DON; DOM-1) is the product of GI tract bacterial action [15,16]. Like DON, DOM-1 is subject to phase II metabolism to glucuronides [6], sulfonates [14] and sulfates [12]. Polygastric animals (i.e., ruminants) and poultry have a high concentration of microflora present both before (i.e., croup, rumen) and after the small intestine (where DON is absorbed). Thus they are better able to produce DOM-1 than monogastric animals such as swine, rodents and humans [2,6,17,18]. DOM-1 formation in humans is reported to be very low or undetectable [19]. One study found that only one of five human fecal samples incubated with DON was able to form DOM-1 [20].

DON evokes toxicity in experimental animals by inducing the release of enteroendocrine hormones that cause emesis [21] and feed refusal [22]. DON is also a potent elicitor of innate immune and proinflammatory responses through activation of mononuclear phagocytes [23,24]. Recently, it was reported that male mice are more sensitive than female mice to induction of food refusal and innate immune system activation by 1 mg/g bw DON [25]. Higher anorectic and proinflammatory cytokine responses to DON in male mice might result from slower metabolism and/or elimination of the toxin. The goal of this study was to test the hypothesis that the capacity of the mouse to metabolize and excrete DON is sex-dependent. To address this hypothesis, we compared male and female mice with respect to (1) clearance rate in vivo and metabolite profiles in excreta and tissues after a single intraperitoneal (i.p.) challenge with 1 mg/kg bw DON and (2) capacity of the liver and kidney microsomes to glucuronidate DON ex vivo. The results presented herein indicate that male mice excrete DON more slowly than female mice, but that these differences in excretion are not likely due to reduced capacity to glucuronidate or otherwise metabolize DON.

## 2. Results

### 2.1. Urinary and Fecal Excretion of DON and DON Metabolites Differ by Sex

**Figure 2:**
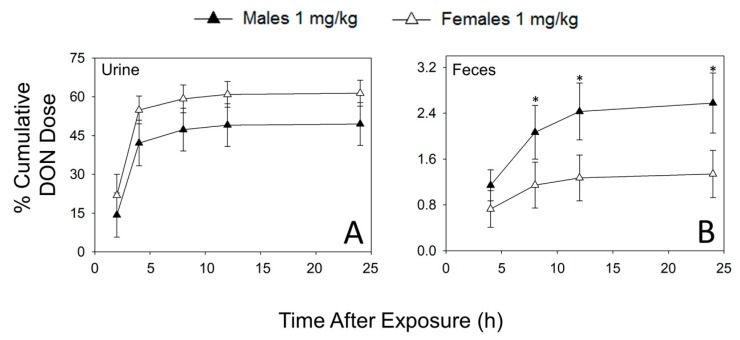
Total DON excretion is slower in male mice than female mice (ELISA analysis). (**A**) Urinary excretion, (**B**) fecal excretion. Data are mean ± SEM (*n* = 8/gp). Asterisks indicate statistical significance from female at time point (*p* < 0.05).

Following i.p. administration of 1 mg/kg bw DON, toxin excretion in urine and feces by male and female mice were compared over 24 h post-injection (PI) using an ELISA that was cross-reactive with DON-3-GlcA. Males exhibited slower clearance rates of DON equivalents than females (Figure 2A). At 2 h PI, female mice had excreted 22% of the DON dose while males had excreted only 14%. By 8 h PI, the majority of the recoverable dose had been excreted in the urine of males and females, accounting for 49% and 60% of the total dose, respectively.

Interestingly, between 8 h and 24 h PI, male mice excreted significantly more of the cumulative DON dose in the feces than females (Figure 2B); however, this only accounted for a small portion of the total recovered dose (2.5 and 1.2 percent, respectively). Upon study termination (24 h PI), ELISA revealed that 63% of the total dose had been recovered in female excreta (62% in urine, 1.3% in feces) and 52% of the total dose had been recovered in male excreta (49% in urine, 2.6% in feces).

Because ELISA likely lacks specificity for some DON metabolites, LC–MS/MS was used to characterize and quantify DON metabolites recoverable in urine and feces over 24 h from male and female mice. Like ELISA, LC–MS/MS analysis revealed that male mice had a lower DON clearance rate than female mice. Mean recoveries of DON plus its metabolites in urine over the 24 h study period were 79 percent for males and 90 percent in females. At 2 and 4 h PI, DON was the major species excreted in urine with concentrations in females being 84 and 53 percent higher, respectively than those observed in males (Table 1). At 24 h, DON was recovered at 37 and 55 percent of the total dose for males and females, respectively. DON-3-GlcA was the major metabolite detectable in urine during the first 8 h with percent total DON dose being 28 and 22 for males and females, respectively. DON-8,15-hk-8-GlcA, and iso-DON-8-GlcA accumulated up to 8 h at concentrations that were approximated 30 percent and 10 to 16 percent, respectively, of those for DON-3-GlcA. Both DON-3- and DON-15-sulfate were also found in female urine accounting for 1.2 percent of the total DON dose at 24 h. Interestingly, these latter two metabolites were undetectable in the urine of males.

Like ELISA, LC–MS/MS indicated that male mice eliminated twice as much DON via feces as females over a 24 h period. Mean recoveries of DON and its selected metabolites in feces over the total study period were 8.3 percent for males and 4.1 percent in females (Table 2). DON S2 was the major species detected in feces, accounting for 4.9 and 1.9 percent of total dose in males and females, respectively. This was followed by DON with percentage of total dose being 2.5 in males and 1.2 in females. Finally, DON S1 and S3 were also detectable in feces of males and females but these accounted for less than 1 percent of the total DON dose.

### 2.2. Hepatic and Renal Levels of DON Metabolites Differ by Sex

Major DON species present in livers and kidneys from male and female mice were compared by LC–MS/MS. DON and its metabolites were largely absent by 8 h in these organs. DON-3-GlcA was the major metabolite present at 1 h in liver with concentrations being twice as much in males than females (Table 3). DON was present at equivalent concentrations in male and female livers. A second metabolite, DON-8,15-hk-8-GlcA, was also evident at 1 and 2 h in males at twice the levels of that in females. By 4 h most DON, DON-3-GlcA and DON-8,15-hk-8-GlcA were cleared. Finally, it was notable that, consistent with the observations in urine, small concentrations of DON-3- and DON-15-sulfates were detectable in the livers of females, but not males.

DON and its metabolites were present in kidney over the first 2 h, but declined significantly by 4 h (Table 4). DON concentrations were largely similar in males and females. DON-3-GlcA was the major metabolite present with concentrations being three times more in males than females. DON-8,15-hk-8-GlcA, was also evident in males at approximately 3 to 4 times the levels of those in females. While lower than the aforementioned metabolites in kidney, iso-DON-8-GlcA was five or more times higher in males at 1 and 2 h and was detected only in males at 4 h.

### 2.3. Ex Vivo DON Glucuronidation Rates by Microsomes Differ by Sex

The effects of sex on DON glucuronidation by hepatic and renal microsomal enzymes were compared (Table 5). In liver microsomes from mice, the rank order for glucuronide formation was DON-3-GlcA > DON-15-GlcA > i-DON-8-GlcA ≈ DON-hk-GlcA ≈ i-DON-3-GlcA. The rate of DON-3-GlcA formation was higher in microsomal incubation mixtures prepared from males than females. Upon incubation with DON, renal mouse microsomes from either sex were incapable of generating glucuronide metabolites. Interestingly, human hepatic microsomes from male and females produced DON-15-GlcA at equivalent rates and the same held true for DON-3-GlcA.

### 2.4. Hepatic UDP-GA Concentrations Do Not Differ by Sex

**Figure 3:**
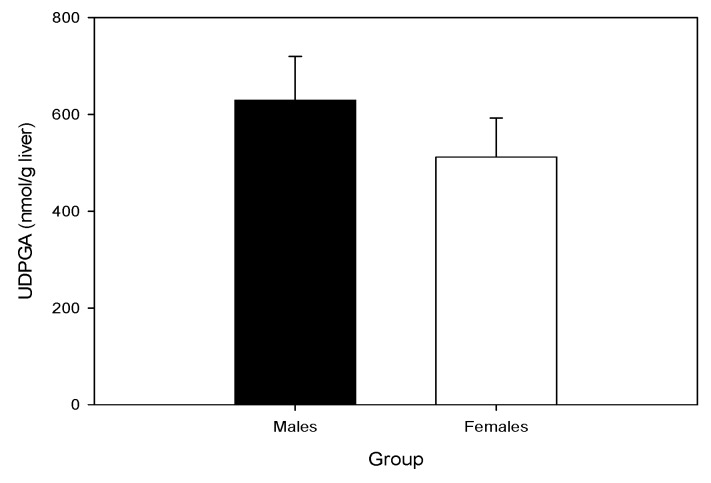
Comparison of murine hepatic UDP-GA concentrations by sex. Data are mean ± SEM (*n* = 10/gp).

Endogenous uridine 5′-diphosphoglucuronic acid (UDP-GA) could potentially be a limiting factor for the glucuronidation rate in vivo. When the concentrations of UPD-GA were measured in the livers of mice, sex differences were not evident (Figure 3). The concentration observed were in agreement with the UDP-GA levels previously reported in mice [26].

## 3. Discussion

Based on the prior observation that DON-induced feed refusal and cytokine production in mice are sex-dependent [25], we compared here the metabolism and excretion of this mycotoxin in male and female mice. Two major findings were made. First, intact DON was excreted more rapidly in female than male mice. Second, these differences could be not be linked to reduced capacities of male mice to glucuronidate DON in vivo or in vitro.

It should be emphasized that intraperitoneal administration was chosen here to replicate the route of exposure used in the aforementioned toxicology study in mice [25]. DON administered by this route is likely absorbed primarily through the portal circulation and, therefore, passes through the liver before reaching other organs. This approach therefore precluded potential metabolism to DOM-1 by the microbiota in the rodent gut and more closely reflects humans which do not exhibit DOM-1 or its metabolites in urine [19]. It should be noted that DOM-1 or its phase II metabolites were not detected in this study to an appreciable extent. This likely reflects the use of i.p. administration which bypasses metabolism by GI microflora to DOM-1. Human GI tract microbiota are generally incapable of metabolizing DON to DOM-1, while rodent microflora is capable of this type of metabolism [2,20,27,28]. Thus, i.p. exposure should more closely mimic human metabolism.

A previous comparison of DON excretion by sex in rats also reported lower recovery of DON and DON metabolites in urine of male rats compared with female rats [11]. When orally gavaged daily with 0.5 mg/kg bw DON for 5 days, 57% of the total DON dose was eliminated in the urine of female rats, while only 27% of the total DON dose was recovered in the urine of similarly treated male rats. This difference was also evident when rats were gavaged with 2.5 mg/kg bw DON with female rats and male rats excreting 42% and 24%, respectively, of the total DON dose via urinary elimination. Consistent with the rat, we found that following a single i.p. dose of DON, male mice excreted 11% less of the total dose in the urine than females over the duration of the study.

It is notable that higher fecal elimination of DON corresponded to its slower urinary elimination. This could indicate that greater enterohepatic cycling is occurring in male mice when compared to female mice, which would prolong toxin elimination in these groups. It should be noted, however, that fecal DON and metabolites account for only a small portion of the total recovered dose.

Our results comparing mouse and human hepatic microsomal glucuronidation of DON are consistent with previous reports of species-dependent DON glucuronide formation. DON-3-GlcA is the major DON glucuronide metabolite found in mouse urine [2]. DON-3-GlcA is also shown to be the predominant glucuronide formed by mouse hepatic microsomes treated with DON [9,10]. In humans, DON-15-GlcA is the major glucuronide metabolite formed, with DON-3-GlcA being a minor metabolite found in both urine samples and ex vivo microsomal treatments with DON [4]. Additionally, our observation that ex vivo rates of DON glucuronide formation were greater in murine hepatic microsomes mice than those of human are also consistent with previous studies [9,10].

Besides glucuronidation, sulfonation of DON has been demonstrated in rats [11,12,14,29]. Differential formation of the DON sulfonates by sex could be another contributing factor to slower excretion rates observed in males compared to female mice. However, DON sulfonates were excreted in feces of males at twice the rate of females. Furthermore, sulfonates were not detectable in urine in this study.

Why is there more unconjugated DON in the urine of female mice? One possible explanation is that female C57BL6 mice innately drink more water and produce more urine than males when adjusted for body weight [30]. Thus, females might excrete DON more rapidly due to higher urine production. A more likely possibility is that slower urinary excretion in male mice is the result of lower capacity to transport conjugated metabolites in the liver and kidney. In support of this contention, female mice have been reported to greater expression of drug efflux transporters responsible for excretion of conjugated compounds (i.e., glucuronides, sulfates) than male mice. These include P-glycoprotein and multiple resistance-associated protein 2 (MRP2) [31] and MRP3 and MRP4 [32]. Indeed administration of testosterone decreases expression of hepatic P-glycoprotein and MRP2 in mice [31]. Further consistent with this notion, increased concentrations of DON metabolites were observed here in livers and kidneys of male mice as compared to female mice. Further experimentation on the involvement of MRPs in sex-dependent DON clearance is thus warranted.

## 4. Conclusions

DON-induced anorectic effects and proinflammatory cytokine responses are much greater in male than female mice [25]. Increased sensitivity of male mice to DON’s toxic effects as compared to females corresponds to decreased ability to eliminate the toxin via urine but does not appear to relate to the few minor differences in metabolism that were found in this study. Understanding the mechanisms for these observations, particularly the role of drug efflux transporters, will be critical in fine-tuning of existing risk assessments for DON and its congeners.

## 5. Experimental

### 5.1. Animals

The Institutional Animal Care and Use Committee at Michigan State University approved all animal experiments (AUF No. 01/14-006-0) prior to their initiation. Adult male and adult female C57BL6 mice (12 weeks old) were purchased from Charles River Laboratories (Portage, MI, USA). Mice were housed singly under a 12 h light/dark cycle, with constant temperature (21–24 °C) and humidity (40–55%). Mice were housed in polycarbonate cages with aspen bedding in all experiments, except when metabolism cages were used. In the experiments involving metabolism cages, mice were rested 1 week after arrival in polycarbonate cages and then transferred to metabolic mouse cages (Techinplast, West Chester, PA, USA). All mice were acclimated to high fat pellet diet (45% kcal from fat; Research Diets, Inc., New Brunswick, NJ, USA) for 1 week prior to DON exposure or sacrifice to maintain consistency among experiments. High fat diet was verified to be free of DON using the ELISA protocol described below. Since the semi-purified diet employed here did not contain DON, neither the toxin nor its metabolites were detectable in excreta and tissues of naïve animals. Therefore data were not shown for vehicle-treated animals in the results.

### 5.2. Chemicals and Reagents

DON used for i.p. injections and microsomal incubations was obtained from Dr. Tony Durst (University of Ottawa, ON, Canada) and purity was verified to be ≥98% by elemental analysis (Galbraith Labs, Knoxville, TN, USA). UDP-GA and alamethicin (from *Trichoderma viride*) were purchased from Sigma-Aldrich (St. Louis, MO, USA). 3-hydroxybenzo[a]pyrene was purchased from Toronto Research Chemicals, Inc. (Toronto, ON, Canada). DON sulfonates 1, 2 and 3 were produced as described in [33] and production of DON- and iso-DON glucuronides is outlined in [6].

### 5.3. Experimental Designs

The effects of sex on urinary and fecal excretion of DON metabolites following exposure of 1 mg/kg bw DON via intraperitoneal injection was compared using Nalgene metabolic cages (MTB-0311; Nunc-Nalgene, Rochester, NY, USA). After acclimation to metabolism cages for 1 week, mice (*n* = 8/gp) were fasted from 10:00 AM to 6:00 PM, exposed to DON, and food replaced. DON was dissolved in sterile physiological saline to equal 1 mL injections based on preliminary studies revealing that this injection volume was found to facilitate early urine collection time points. Urine and fecal sample collection tubes were kept on ice during the duration of the experiment. Urine samples were collected at 2, 4, 8, 12, and 24 h post injection by thoroughly rinsing collection surface with 3 mL distilled water. This process was repeated 3 times with the same 3 mL of water at each time point. Fecal samples were collected at 4, 8, 12, and 24 h PI. Fecal samples were not collected at 2 h PI due to variable and low sample amount. Samples were stored at −80 °C immediately after collection until analysis. Feces were suspended in 0.01 M phosphate buffered (pH 7.5) saline (PBS) (1:5, *w*/*v*) for 10 min at room temperature and then homogenized. Urine and fecal homogenates were then centrifuged at 15,000× *g* for 10 min at 4 °C. Supernatant was removed and then heated at 100 °C for 5 min. Sample were centrifuged again at 15,000× *g* for 10 min at 4 °C and resulting supernatant was used for analysis.

The effects of sex on DON metabolites in liver and kidney were assessed after acute i.p. exposure to the toxin. Groups of male and female mice (*n* = 5–6/group), were fasted as described above and then exposed to 1 mg/kg bw DON in PBS or PBS vehicle via i.p. injection. Cohorts of mice were euthanized via CO_2_ chamber at 1, 2, and 4 h post exposure without food replacement. Liver and kidney were collected, immediately snap frozen, and stored at −80 °C until analysis. Prior to analysis, organs were homogenized 1:1 in PBS. Tissue homogenates were heated at 100 °C for 5 min and then centrifuged at 14,000× *g* for 10 min at 4 °C. The resulting supernatant was analyzed for DON and its metabolites by LC–MS/MS.

### 5.4. ELISA of Urine and Feces

Urinary and fecal DON equivalent concentrations were measured using a DON Veratox HS ELISA with modifications as previously described [34]. Key adjustments to increase sensitivity included dilution of enzyme conjugate, increased incubation time and inclusion of after-market DON standards ranging from 1 to 200 ng/mL. DON was reported as DON equivalents because this ELISA was 100% cross-reactive with DON-3-GlcA, the major glucuronide DON metabolite in the mouse [35]. Cross-reactivity with other DON glucuronides was not determined because of lack of available standards.

### 5.5. LC–MS/MS Analysis of Excreta and Tissues

LC–MS/MS analyses were carried out on an Agilent 1290 series UHPLC system coupled to a 6500+ QTrap mass spectrometer equipped with an IonDrive Turbo V^®^ source (Sciex, Foster City, CA, USA). Analytes were separated in gradient elution on a Kinetex biphenyl column (150 × 3 mm, 2.6 µm, Phenomenex, A Schaffenburg, Germany) at 30 °C using water and acetonitrile, both containing 0.1% (*v*/*v*) acetic acid, as mobile phases A and B, respectively. The gradient started with an isocratic hold time at 5% B for 0.5 min. Then, the percentage of B was linearly increased to reach 22% at 8.5 min. The column was subsequently washed at 100% B from 9.0 to 10.9 min. Finally, the column was re-equilibrated at 5% B for 2.5 min, reaching a total run-time of 13.5 min. The flow rate was 0.4 mL/min and the injection volume was 3 µL. The LC-stream was directed into the mass spectrometer between 2.0 and 9.5 min.

Tandem mass spectrometric analysis started with electrospray ionization in the negative ionization mode. Parameters of selected reaction monitoring transitions (dwell time of 15 ms) are given in Table 6. Ion source settings were: curtain gas 35 psi, source temperature 400 °C, nebulizer gas (GS1) 60 psi, heater gas (GS2) 40 psi, ion spray voltage −4500 V. Analyst^®^ software version 1.6.3 (Sciex, Foster City, CA, USA) was used for instrument control and data analysis.

Analytes were quantified on the basis of neat solvent calibration functions established between 0.3 and 300 ng/mL for all compounds (except DON and DON-3-GlcA: 0.3 to 1000 ng/mL). The concentrations of DON-8,15 hk-8-GlcA and iso-DON-8-GlcA for which no standard was available at that time were estimated on the basis of the DON-3-GlcA quantifier transition (*m*/*z* 471.1 -> 113.1). Limits of detection (LODs) were assessed at a signal to noise ratio equal to 3 (see Table 6). Limits of quantification (LOQs) were assessed at signal to noise ratios equal to 10 and therefore either 3.3 times higher than LODs or equal to the concentration of the lowest calibration standard (0.3 ng/mL) for analytes the LOD of which was <0.1 ng/mL. When responses fell between LOQ and LOD, data were reported as the LOQ/2.

The Kinetex biphenyl column was chosen because DON-3-sulfate and DON-15-sulfate could be almost baseline separated under the used conditions. However, on this column, DON-3-GlcA co-elutes with DON-15-GlcA. To verify if DON-15-GlcA is present in the samples, the SRM transition *m/z* 471.1 -> 441.1 that is specific for DON-3-glucuronide was additionally evaluated and the intensity ratio of the transitions to *m*/*z* 441.1/113.1 was calculated. In all samples, the intensity ratio was the same as in the standards, indicating absence or minor formation of DON-15-GlcA in mouse urine and tissue samples.

### 5.6. Hepatic and Renal Microsomal Conversion of DON to DON Glucuronides

Effects of sex and species on DON metabolism by microsomal enzymes were compared. Hepatic and renal microsomes from mouse (*n* = 6/gp) were prepared by tissue homogenization and differential centrifugation according to Lake [36]. Microsomal protein content was determined using the Pierce BCA Protein Assay kit (Life Technologies; Grand Island, NY, USA). Male and female human liver microsomes (pool of 10 individuals each) were obtained from Xenotech (Lenexa, KS, USA). Conditions of microsomal incubations were as previously reported [9]. Reaction mixtures (200 μL) contained: 100 mM potassium phosphate buffer (pH 7.4); 5 mM MgCl_2_; 2.5 mM UDP-GA; 25 μg/mL alamethicin; 1 mg/mL microsomal protein; and 4 μL of either 5 μM or 20 μM DON in methanol/water 50/50 (*v*/*v*). Reactions were preincubated at 37 °C for 5 min. UDP-GA was then added to initiate reaction and tubes further incubated at 37 °C for 60 min. The addition of 200 μL ice-cold acetonitrile terminated the reaction and tubes were centrifuged at 14,000× *g* for 5 min. A supernatant aliquot (200 μL) was then evaporated to dryness in a vacuum concentrator. Samples were analyzed by LC–MS/MS on the 6500+ QTrap system described above, using chromatographic conditions allowing baseline separation of DON-3-GlcA and DON-15-GlcA as described in Schwartz-Zimmermann et al. [6].

### 5.7. Determination of Hepatic UDP-GA Concentrations

Hepatic UDP-GA concentrations were determined by UDP-GA dependent formation of 3-hydroxybenzo[a]pyrene glucuronide as described in a prior study [37]. Mice (*n* = 10/gp) were fasted from 10:00 AM to 6:00 PM as in previous studies comparing sex and age differences in tissue DON concentrations. Mice were humanely euthanized by anesthetization with isoflurane and then cervical dislocation. Small pieces of liver (approx. 50 mg) were quickly excised and immediately dropped into 5 mL of boiling distilled water for 3 min. Tissues were cooled on ice and then dounce homogenized in sterile water. Homogenized samples were centrifuged at 3000× *g* for 10 min at 4 °C. Supernatant was removed and stored at −80 °C until analysis. The reaction mixture (200 μL) contained: 20 μmol Tris buffer (pH 7.6); 1 μmol MgCl_2_; 0.01% Brij-58 (*w*/*v*); 10 nmol of 3-hydroxybenzo[a]pyrene in 10 μL of methanol; 50 μL liver tissue homogenate; and 50 μg of guinea-pig liver microsomal protein (Xenotech, Lenexa, KS, USA). Reaction tubes were held on ice until incubation at 37 °C in a water bath with mild shaking for 30 minutes. To terminate reaction, reaction mixtures were added to 6 mL of chloroform/methanol (2:1, *v*/*v*) and and 0.8 mL of distilled water and vigorously shaken. Tubes were centrifuged at 1000× *g* for 1 min. 200 μL of the aqueous-methanol phase was added to black 96-well plates. Fluorescence of benzo[a]pyrene glucuronide end product was measured at 378 nm excitation/425 nm emission. UDP-GA amounts were determined from a standard curve of known UDP-GA concentrations. Samples were analyzed in duplicates.

### 5.8. Statistical Analysis

All statistical analyses were conducted using SigmaPlot version 11.0 (Jandel Scientific; San Rafael, CA, USA). Statistical comparisons by sex at a specific time point were made by a Student’s *t*-test, unless normality failed in which case a Mann-Whitney Rank Sum test was performed. A one-way analysis of variance (ANOVA) was used to determine statistical significance between sex in experiment comparing DON glucuronidation by human liver microsomes. Differences were considered significant when *p* < 0.05.

## Figures and Tables

**Table 1:** Urinary excretion of DON and DON metabolites profiles differs by sex. DON (1 mg/kg bw) was administered i.p. to mice. Urine was collected and analyzed by LC–MS/MS.

	% Cumulative DON Dose									
Time	2 h		4 h		8 h		12 h		24 h	
Sex	♂	♀	♂	♀	♂	♀	♂	♀	♂	♀
**DON**	11.1 ± 0.4	20.4 ± 7.5	32.9 ± 7.0	50.4 ± 4.6	35.9 ± 6.9	53.8 ± 4.5 *	37.1 ± 6.9	54.9 ± 4.3 *	37.3 ± 6.9	55.2 ± 4.3 *
**DON-3-GlcA**	7.6 ± 4.8	7.1 ± 2.7	25.0 ± 4.9	20.0 ± 2.2	27.9 ± 4.4	21.5 ± 2.2	28.9 ± 4.4	22.0 ± 2.1	29.1 ± 4.4	22.1 ± 2.1
**DON-8,15-hk-8-GlcA**	2.2 ± 1.4	2.4 ± 0.9	7.4 ± 1.4	6.7 ± 0.7	8.3 ± 1.3	7.3 ± 0.7	8.6 ± 1.3	7.4 ± 0.7	8.7 ± 1.3	7.4 ± 0.7
**iso-DON-8-GlcA**	0.9 ± 0.6	1.1 ± 0.4	3.1 ± 0.6	3.1 ± 0.3	3.6 ± 0.6	3.5 ± 0.3	3.9 ± 0.6	3.6 ± 0.3	4.0 ± 0.6	3.7 ± 0.3
**DON-3-sulfate**	ND	0.2 ± 0.1 *	ND	0.5 ± 0.1 *	ND	0.5 ± 0.1 *	ND	0.5 ± 0.1 *	ND	0.6 ± 0.1 *
**DON-15-sulfate**	ND	0.2 ± 0.1 *	ND	0.6 ± 0.1	ND	0.6 ± 0.0	ND	0.6 ± 0.1 *	ND	0.6 ± 0.1 *
**Total**	21.7 ± 13.1	31.1 ± 11.5	68.4 ± 13.9	81.2 ± 7.8	75.7 ± 13.1	87.2 ± 7.6	78.5 ± 13.2	89.1 ± 7.3	79.1 ± 13.1	89.6 ± 7.2

Data are mean ± SEM (*n* = 8). Values for DON-8,15-hk-8-GlcA and iso-DON-8-GlcA are estimated based on the peak areas of the DON-3-GlcA quantifier transition. Asterisk indicates significant difference from male at time point (*p* < 0.05).

**Table 2:** Fecal excretion of DON and DON sulfonate metabolites profiles differs by sex.

	% Cumulative DON Dose							
Time	2 h		4 h		8 h		12 h	
Sex	♂	♀	♂	♀	♂	♀	♂	♀
**DON**	1.10 ± 0.24	0.68 ± 0.29	1.90 ± 0.41	1.00 ± 0.35 *	2.32 ± 0.45	1.15 ± 0.36 *	2.48 ± 0.49	1.21 ± 0.37 *
**DON S1**	0.04 ± 0.01	0.02 ± 0.01	0.29 ± 0.07	0.19 ± 0.03	0.62 ± 0.05	0.38 ± 0.03 *	0.85 ± 0.08	0.47 ± 0.02 *
**DON S2**	0.16 ± 0.05	0.05 ± 0.02 *	1.56 ± 0.43	0.73 ± 0.15	3.46 ± 0.40	1.51 ± 0.15 *	4.93 ± 0.57	1.93 ± 0.16 *
**DON S3**	0.03 ± 0.01	0.01 ± 0.01	0.22 ± 0.09	0.13 ± 0.03	0.29 ± 0.10	0.15 ± 0.03	0.35 ± 0.04	0.17 ± 0.03
**Total**	1.34 ± 0.21	0.75 ± 0.31	3.93 ± 0.84	2.12 ± 0.43	6.70 ± 0.86	3.32 ± 0.44	8.30 ± 1.05	4.05 ± 0.43

DON (1 mg/kg bw) was administered i.p. to mice. Feces was collected over 24 h and analyzed by LC–MS/MS. Data are mean ± SEM (*n* = 8). Asterisk indicates significance from male at time point (*p* < 0.05).

**Table 3:** Comparison of DON and DON metabolite equivalent concentrations in liver by sex.

	Concentration (nmol/g)					
Time	1 h		2 h		4 h	
Sex	♂	♀	♂	♀	♂	♀
**DON**	1.31 ± 0.15	1.76 ± 0.16	0.55 ± 0.09	0.59 ± 0.05	0.10 ± 0.01	0.09 ± 0.01
**DON-3-GlcA**	3.00 ± 0.35	1.45 ± 0.18 *	1.10 ± 0.22	0.50 ± 0.10 *	0.63 ± 0.30	0.12 ± 0.04 *
**DON-8,15-hk-8-GlcA**	0.94 ± 0.13	0.46 ± 0.06 *	0.33 ± 0.05	0.15 ± 0.02 *	0.17 ± 0.08	0.03 ± 0.01
**iso-DON-8-GlcA**	0.22 ± 0.03	0.09 ± 0.02 *	0.09 ± 0.02	0.03 ± 0.01 *	0.08 ± 0.03	0.02 ± 0.01
**DON-3-sulfate**	ND	0.02 ± 0.003 *	ND	0.01 ± 0.004 *	ND	ND
**DON-15-sulfate**	ND	0.02 ± 0.004 *	ND	0.02 ± 0.003 *	ND	ND

DON (1 mg/kg/bw) was administered i.p. to mice. Tissues were collected over 4 h and analyzed by LC–MS/MS. Values for DON-8,15-hk-8-GlcA and iso-DON-8-GlcA are estimated based on the peak areas of the DON-3-GlcA quantifier transition. Data are mean ± SEM (*n* = 8); ND = not detected. Asterisk indicates significance from male at time point (*p* < 0.05).

**Table 4:** Comparison of DON and DON metabolite concentrations in kidney by sex.

	Concentration (nmol/g)					
Time	1 h		2 h		4 h	
Sex	♂	♀	♂	♀	♂	♀
**DON**	2.09 ± 0.14	1.76 ± 0.16	0.63 ± 0.08	0.59 ± 0.05	0.16± 0.01	0.09 ± 0.01 *
**DON-3-GlcA**	2.86 ± 0.31	0.98 ± 0.09 *	0.99 ± 0.12	0.33 ± 0.04 *	0.17 ± 0.04	0.06 ± 0.01 *
**DON-8,15-hk-8-GlcA**	2.33 ± 0.27	0.74 ± 0.03 *	0.93 ± 0.11	0.21 ± 0.02 *	0.15 ± 0.05	0.05 ± 0.01
**iso-DON-8-GlcA**	0.43 ± 0.04	0.08 ± 0.02 *	0.19 ± 0.02	0.02 ± 0.01 *	0.06 ± 0.02	ND

DON (1 mg/kg/bw) was administered i.p. to mice. Tissues were collected over 4 h and analyzed by LC–MS/MS. Data are mean ± SEM (*n* = 8); ND = not detected. Asterisk indicates significance from male at time point (*p* < 0.05).

**Table 5:** Mouse and human liver microsomes catalyze DON glucuronidation in a sex-specific manner.

			DON Glucuronide Formation (pmol/min/mg protein)				
Sex	Species	DON (µM)	DON-3-GlcA	DON-15-GlcA	iso-DON-8-GlcA	iso-DON-3-GlcA	DON-8,15 -hk -8-GlcA
**♂**	Mouse	5	529 ± 239	72 ± 3	37 ± 1	33 ± 8	33 ± 8
**♀**	Mouse	5	279 ± 269 *	35 ± 0 *	18 ± 0 *	18 ± 0	18 ± 0
**♂**	Mouse	20	1760 ± 309	210 ± 29	145 ± 22	108 ± 19	114 ± 17
**♀**	Mouse	20	1068 ± 31	212 ± 4	125 ± 5	90 ± 5	84 ± 4
**♂**	Human	5	15 ± 15	35 ± 0	ND	ND	ND
**♀**	Human	5	ND	35 ± 0	ND	ND	ND
**♂**	Human	20	44 ± 0	224 ± 2	ND	ND	ND
**♀**	Human	20	44 ± 0	231 ± 14	ND	ND	ND

Data are mean ± SEM (*n* = 3/rep). Asterisk indicates significance from male at treatment level (*p* < 0.05).

**Table 6:** Optimized SRM parameters on the 6500+ QTrap LC–MS/MS system. Product ions are given in the order quantifier (quant) before qualifier (qual) *.

Analyte	Ret. Time (min)	Precursor Ion (*m*/*z*)	Ion Species	DP (V)	Product Ions (*m/z*)	CE (eV)	Ion Ratio (qual/quant)	LOD of Quant ** (ng/mL)
DON	5.85	355.1	[M+CH_3_CO_2_]^−^	−50	59.1/265.1	−38/−18	0.24	0.7
DOM-1	7.01	339.1	[M+CH_3_CO_2_]^−^	−50	59.1/249.1	−40/−17	0.36	0.6
DON-3-sulfate	4.98	375.1	[M−H]^−^	−125	345.1/247.1	−36/−38	0.80	0.2
DON-15-sulfate	4.87	375.1	[M−H]^−^	−110	96.9/247.1	−38/−38	0.06	0.03
DOM-3-sulfate	5.70	359.1	[M−H]^−^	−125	96.9/329.1	−38/−34	0.15	0.1
DOM-15-sulfate	5.83	359.1	[M−H]^−^	−125	96.9/79.9	−28/−118	0.48	0.06
DON-3-GlcA	5.36	471.1	[M−H]^−^	−100	113.1/265.1 *	−35/−38	0.38	0.6
DON-8,15-hk-8-GlcA	4.50	471.1	[M−H]^−^	−100	113.1/265.1	−35/−38	0.12	0.5
i-DON-8-GlcA	2.71	471.1	[M−H]^−^	−100	441.1/113.1	−25/−35	0.47	0.4
i-DON-3-GlcA	5.72	471.1	[M−H]^−^	−100	441.1/113.1	−25/−35	0.13	0.4
DONS 1	2.26	377.1	[M−H]^−^	−130	79.9/331.0	−98/−52	0.04	0.08
DONS 2	2.82	377.1	[M−H]^−^	−105	80.9/79.9	−68/−98	0.15	0.08
DONS 3	3.64	377.1	[M−H]^−^	−130	79.9/347.0	−98/−36	0.40	0.8
DOMS 2	3.93	361.1	[M−H]^−^	−25	80.9/249.1	−65/−30	0.09	0.06

* In addition, the transition to 441.1 (CE −30 eV, ratio transitions to 441.1/113.1:0.20) was monitored; ** Limit of detection of the quantifier transition.
