# Correction: Pestka, J.J., et al. Sex Is a Determinant for Deoxynivalenol Metabolism and Elimination in the Mouse. *Toxins* 2017, *9*, 240

**DOI:** 10.3390/toxins11050238

**Published:** 2019-04-26

**Authors:** James J. Pestka, Erica S. Clark, Heidi E. Schwartz-Zimmermann, Franz Berthiller

**Affiliations:** 1 Department of Food Science and Human Nutrition, Michigan State University, East Lansing, MI 48824, USA; clarkerica7@gmail.com; 2 Center for Integrative Toxicology, Michigan State University, East Lansing, MI 48824, USA; 3 Department of Microbiology and Molecular Genetics, Michigan State University, East Lansing, MI 48824, USA; 4 Christian Doppler Laboratory for Mycotoxin Metabolism, Center for Analytical Chemistry, Department of Agrobiotechnology (IFA-Tulln), University of Natural Resources and Life Sciences, Vienna, 3430 Tulln, Austria; heidi.schwartz@boku.ac.at (H.E.S.-Z.); franz.berthiller@boku.ac.at (F.B.)

The authors wish to make the following corrections to their paper [1].

There is a mistake in the drawing of the structures of iso-deoxynivalenol. The position of the double bond was drawn incorrectly. The correct position is between C8 and C9 as shown in the new Figure 1C.

The changes do not affect the scientific results. The manuscript will be updated and the original will remain online on the article webpage. We apologize for any inconvenience caused to our readers.

## Figures and Tables

**Figure 1:**
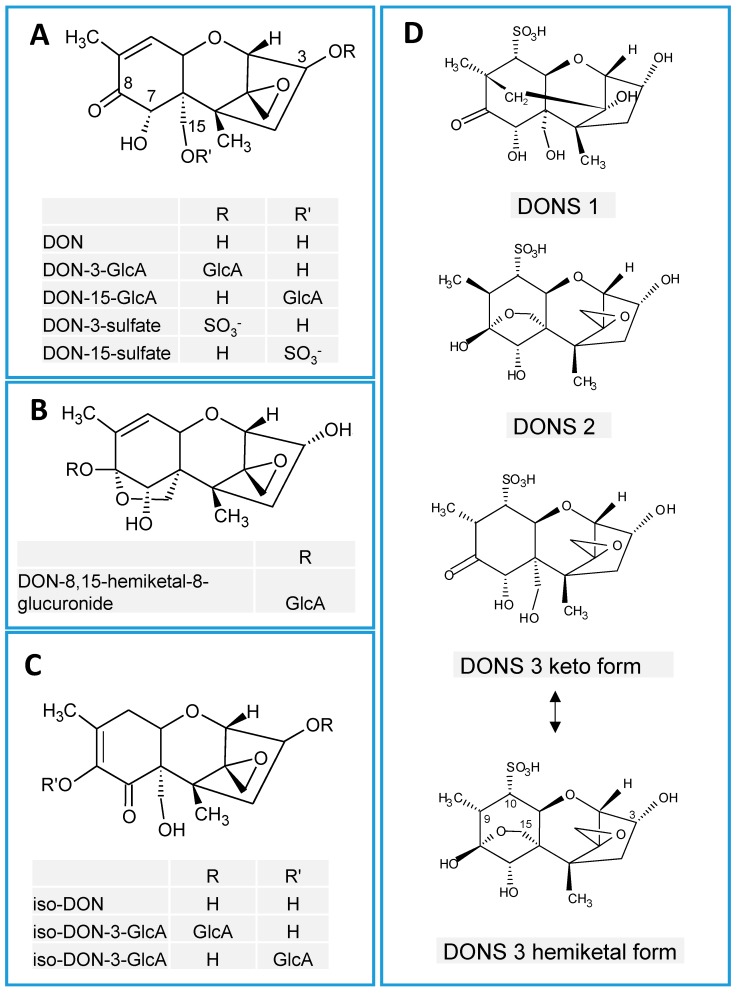
Chemical structures of major deoxynivalenol (DON) metabolites. (**A**) DON-glucuronides (DON-GlcAs) and DON-sulfates, (**B**) DON-8,15 hemiketal-8-glucuronide, (**C**) iso-DON and its glucuronides, (**D**) DON sulfonates (DONS).
